# Predicting Properties of Cyclohexene with Electronic Structure Methods through Adaptive Force Matching

**DOI:** 10.1021/acs.jpcc.6c00465

**Published:** 2026-05-05

**Authors:** Raymond Weldon, Feng Wang

**Affiliations:** Department of Chemistry and Biochemistry, 3341 University of Arkansas, Fayetteville, Arkansas 72701, United States

## Abstract

Electronic structure calculations are powerful tools for determining molecular energies and structures; however, their computational cost limits their application to large systems and hinders the calculation of finite-temperature properties that require ensemble averages. We demonstrated that the Adaptive Force Matching (AFM) method can be used to bridge this gap and predict macroscopic properties of a molecule solely on the basis of electronic structure information. As a proof-of-concept, we focused our work on cyclohexene. By fitting AFM models to reference forces computed using the MP2 and B3LYP-D3 methods, we successfully predicted 16 distinct properties. For all properties with available experimental values for validation, the AFM models achieve very good agreement. For other properties, predictions from AFM models fill gaps in the missing experimental data. While models developed by fitting neat and hydrated phases separately predict the solubility of cyclohexene in outstanding agreement with experimental reference values, a mixed phase model is required for certain properties, such as interfacial tension between cyclohexene and water. The results demonstrate the feasibility and accuracy of using AFM-based molecular dynamics for predicting a wide range of macroscopic properties directly from electronic structure information, opening new avenues for computational materials science and accelerating the discovery and design of novel chemicals.

## Graphical abstract

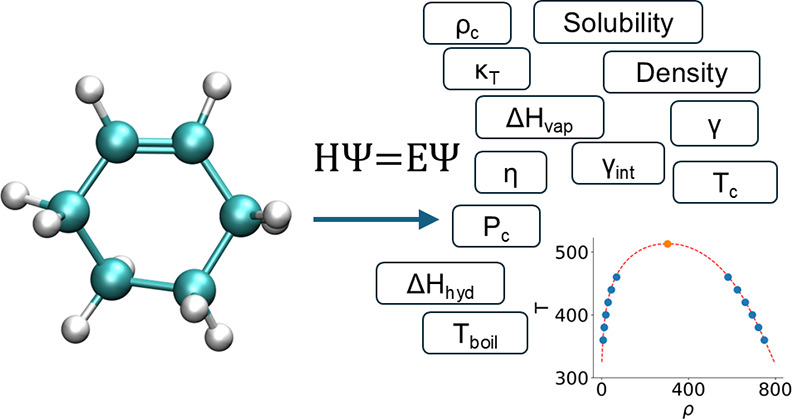

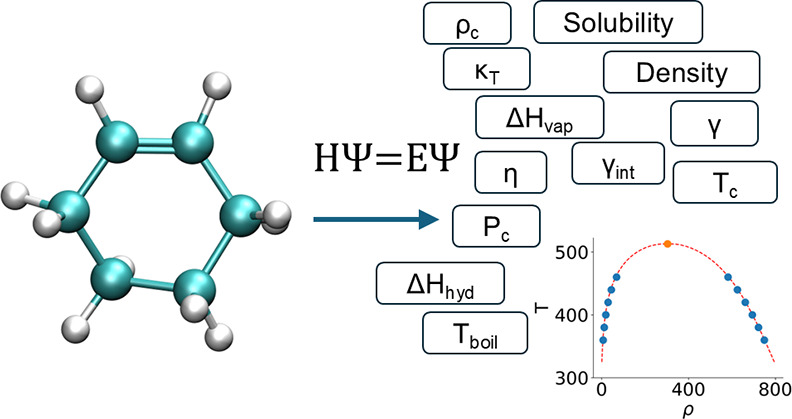

## I Introduction

Electronic structure calculations have emerged as a powerful tool for determining conformational energies and structural information of small molecules.^1–3^ However, accurate electronic structure calculations can become prohibitively expensive for large systems. Even the more computationally efficient density functional theory (DFT) method is typically limited to systems containing no more than a few hundred atoms.^4–6^ A further challenge facing electronic structure methods is that most material properties, such as the free energies, densities, and boiling points of liquids, are ensemble properties, at finite temperatures. To accurately determine these properties, it is necessary to perform ensemble averages, which require more than just information about a few conformations. A much more efficient model is needed for the prediction of such macroscopic properties.

One approach to addressing this challenge is to map quantum mechanical (QM) information onto molecular mechanics force fields.^7–10^ These force fields can then be used in conjunction with methods such as Molecular Dynamics or Monte Carlo Sampling to compute macroscopic properties, enabling the prediction of ensemble properties, at finite temperatures.

While deriving force fields from electronic structure calculations has a long history,^11–16^ the most substantial advances have been made in modeling water,^17,18^ with models such as MB-Pol^19^ and various Adaptive Force Matching (AFM) based approaches^20^ demonstrating good accuracy in predicting macroscopic properties of water.^21–24^ In contrast, the development of purely electronic structure-based models for other materials such as hydrocarbons remains more limited. Although recent AFM models have shown promise in predicting specific properties like density and heat of vaporization,^25,26^ it remains to be seen whether these models, developed by fitting solely to electronic structure calculations, can accurately predict a broad range of physical properties for a given material. The ability to comprehensively predict multiple physical properties by using this approach is still not fully established.

For this proof-of-concept study, we selected cyclohexene as the molecule of interest. As an intermediate in the industrial synthesis of nylon, cyclohexene is a molecule of significant practical importance. Its molecular structure features a double bond, which results in a relatively strained conformation. Furthermore, cyclohexene is characterized by weak intermolecular interactions with dispersion forces dominating its interaction energy. From the perspective of electronic structure-based models, two challenges exist for such dispersion-bound systems. The first challenge lies in the well-recognized difficulty of DFT to accurately model dynamic correlation,^27–29^ which gives rise to dispersion. The other challenge lies in the small cohesion energy of such weakly bound systems; a small 1 kcal error in neat cyclohexene cohesive energy could translate to a large percentage error. Such an error is expected to affect the density prediction, leading to substantial errors in density. For example, for the AFM-based CO_2_ model, minor errors introduced through basis set superposition error led to substantial errors in density.^30^ While AFM has shown good success in modeling polar systems, such as water,^22,23,31^ hydrated ions,^32,33^ and hydrated small molecules,^34,35^ a weakly interacting compound like cyclohexene would make a good test case for evaluating the performance of our approach.

In fact, AFM models for cyclohexene have been created for both neat and aqueous environments.^25^ However, a comprehensive evaluation of the performance of such models for a wide range of properties has not been performed. In this study, we will evaluate the neat model’s ability to predict a range of physical properties, including density (ρ), enthalpy of vaporization (*ΔH*_*vap*_), surface tension (γ), viscosity (η), diffusion constant (*D*_*P*_), isothermal compressibility (κ_*T*_), the free energy of vaporization (*ΔG*_*vap*_), the critical temperature (*T*_*C*_), pressure (*P*_*C*_), and density (ρ_*C*_), and the boiling temperature (*T*_*b*_).

In addition to examining the properties of neat cyclohexene, we also explore the behavior of cyclohexene in a hydrated environment. It is worth noting that the AFM models do not rely on combining rules, which means that the parameters for cyclohexene in a hydrated environment cannot be derived from those of neat cyclohexene and neat water. Instead, a separate model must be developed to describe the behavior of cyclohexene in a hydrated environment. This hydrated model is found to reliably predict the hydration free energy (*ΔG*_*hyd*_), the enthalpy of hydration (*ΔH*_*hyd*_), and the diffusion constant (*D*_*H*_).

By determining both *ΔG*_*hyd*_ and *ΔG*_*vap*_, we predict the solubility of cyclohexene. However, while it is possible to predict solubility by fitting separate models to neat and hydrated cyclohexene, not all properties of the cyclohexene–water mixture can be determined using this approach. For example, determining the interface tension (γ_*int*_) between cyclohexene and water requires a single model that can describe both phases. To address this problem, we developed a mixed-phase model by fitting to both neat and hydrated cyclohexene together. This model enables us to predict γ_*int*_ of the cyclohexene–water mixture, and we also report on its performance in modeling the pure cyclohexene liquid.

## II Computational Details

### A Model Development

AFM has proven to be a powerful tool for mapping advanced electronic structure potential energy surfaces onto efficient force field models.^33,36,37^ AFM usually fits dispersion independently of other nonbonded parameters. By decoupling the relatively weak but important dispersion interaction from the Coulombic force and short-range repulsion, we can develop a more robust model. After the fitting of the dispersion, the AFM procedure iterates through three main steps: the sampling step, the Quantum Mechanics (QM) or QM/Molecular Mechanics (MM) step, and the force matching (FM) step.

In the sampling step, molecular dynamics (MD) simulations are typically performed to generate a training set of conformations that are representative of the conformational space of interest. The QM or QM/MM step involves calculating the forces using a reference electronic structure method, which provides the necessary data for determining the force field parameters. The actual parameter determination is carried out in the FM step, which often involves multiple stages. For example, intermolecular parameters are typically fitted before intramolecular parameters, which reduces the numerical challenges of fitting strongly coupled parameters.

For cyclohexene, AFM models have been previously developed for both the neat and hydrated phases.^25^ The neat phase model was fitted without using partial charges against reference forces computed using the MP2 method, with dispersion interactions calculated using Symmetry-Adaptive Perturbation Theory (SAPT)^38^ at the quality of MP4. While the accuracy of MP2 is important for a weakly interacting system, such as pure cyclohexene, the hydrated phase model was fitted against forces computed using the less computationally demanding B3LYP-D3­(BJ) method.^39–42^ The computational cost of AFM is mostly in QM or QM/MM calculations. The MP2 calculations for the development of the neat model take 45 min each using ORCA on an AMD EPYC 9454 CPU utilizing 12 cores. The B3LYP-D3­(BJ) calculations for the development of the hydrated model take about 15 min each on the same hardware. Approximately 800 such single-point force calculations are needed to generate all of the reference forces for the training of each of the neat and hydrated models. As the development of these models has been previously described, we will provide only a brief summary of the procedures used to develop them in the Supporting Information.

While utilizing two separate models for neat and hydrated cyclohexene enables the determination of a wide range of properties, including the solubility of liquid cyclohexene in water, it does not permit the simulation of a cyclohexene droplet in water or the calculation of the interface tension between the two liquids. To facilitate simulations of mixtures, a single model must be developed that can describe both phases in one simulation.

This requires fitting a model against reference forces from both the neat and the hydrated phases. Notably, the AFM models for cyclohexene liquid and hydrated cyclohexene use different partial charges. As cyclohexene is nonpolar, the previously developed neat cyclohexene model was fit without using partial charges, whereas the hydrated cyclohexene model in water was fit with partial charges. For a model to simulate liquid cyclohexene in contact with liquid water, cyclohexene must use a single set of partial charges. While one possibility is to fit one set of charges that optimize for both the neat-cyclohexene and hydrated cyclohexene, we chose to take a simpler approach: using the partial charges from the hydrated cyclohexene model for the mixed-phase model, as well. We argue that, for a nonpolar system, the actual partial charge is less important, as evidenced by our success in developing such models without partial charges.

Another complication arises from the fact that the neat model employed a different atom typing scheme than that of the hydrated model, with the latter utilizing more atom types, as illustrated in Figure 1. Since the repulsion and Coulombic terms are strongly coupled, two atoms with different partial charges should be fitted with distinct repulsion terms. The decision to forego partial charges in the previous development of the neat model allowed it to use only two atom types for carbon and two types for hydrogen, whereas the hydrated model employed three types of carbon and three types of hydrogen. The mixed model will use the same atom types as the hydrated model.

**Figure 1:**
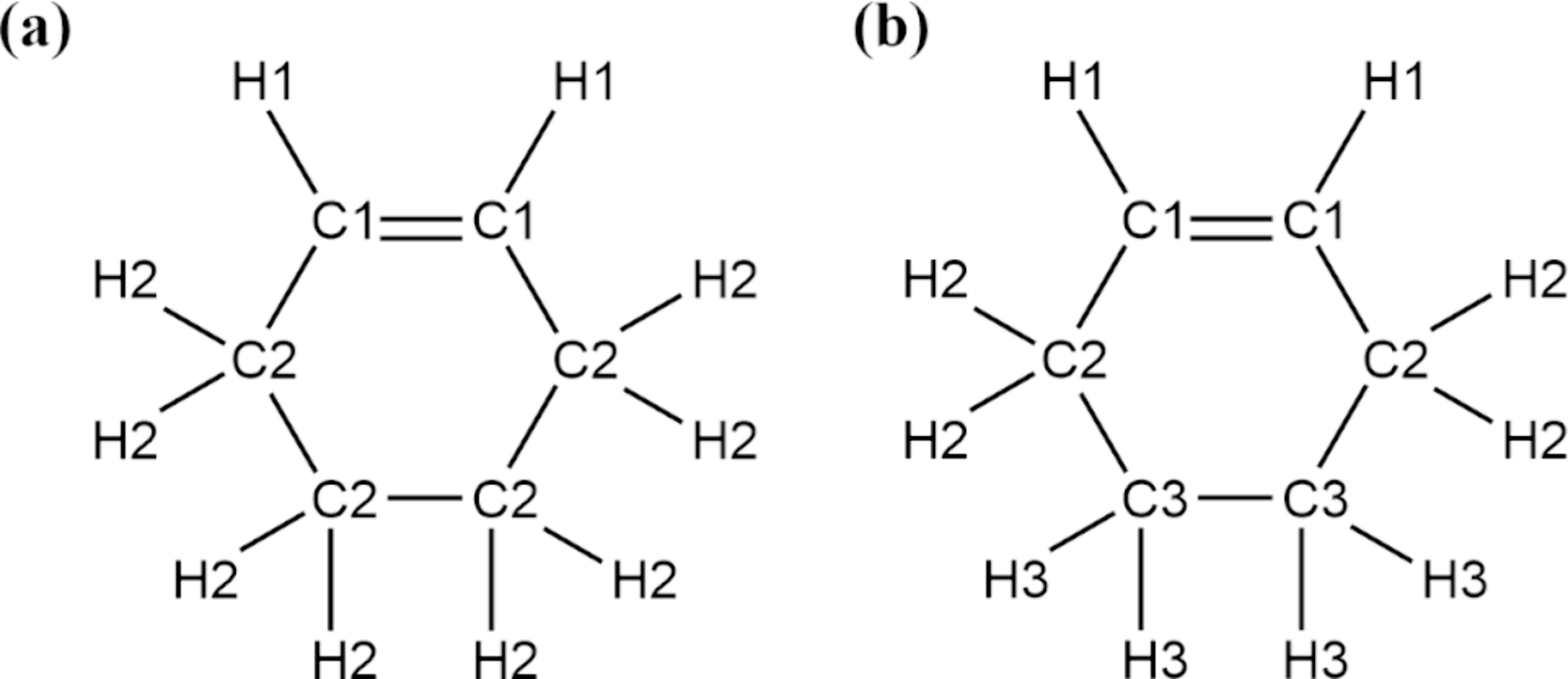
Atom types used for the (a) CHE_n_ (b) CHE_h_ models.

To better understand the performance of the mixed-phase model, we present scatter plots in Figure 2 showing intermolecular forces and torques as well as atomic forces for the individual phase models and the mixed-phase model. For the individual phase models, the neat phase QM references were fit to the neat phase cyclohexene (CHE) model CHE_n_, and the hydrated phase QM/MM references were fit to the hydrated model CHE_h_. The mixed-phase model, CHE_m_, is fitted to reference calculations from both phases. A perfect fit is a straight line along the diagonal. It is clear from Figure 2 that no discernible difference in fit quality can be seen when comparing the individual and mixed models.

**Figure 2:**
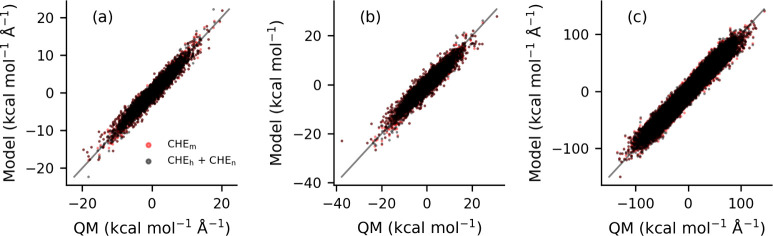
Scatter plot comparing the performance of the models to the reference QM forces and torques being fit. (a) Molecular force, (b) molecular torque, and (c) atomic force are shown for the CHE_m_ model (red) and CHE_h_ and CHE_n_ models (black).

The root-mean-square errors (RMSE) for the two types of models are reported in Table 1, along with the slope and *R* ^2^ values for the linear regression between the fitted and reference forces. The RMSE values for molecular force and torque reflect the models’ ability to reproduce the intermolecular component of the reference forces. Interestingly, the CHE_m_ model performs approximately 2% better than using the individual models for reproducing both the molecular force and torque. With AFM, the nonbonded short-range parameters for cyclohexene–water and cyclohexene–cyclohexene interactions are independent. Therefore, adding neat phase reference data to the training set will not affect the nonbonded cyclohexene–water parameters, as the CHE_m_ model employs the same partial charges as CHE_h_. Consequently, the two models yielded identical cyclohexene–water interactions. The observed slight improvement in intermolecular RMSE’s is attributable to the improved reproduction of intermolecular interactions within the neat phase. This improvement suggests that employing partial charges, even those fitted in an aqueous environment, can lead to slightly better model performance compared with a model without them.

**Table 1:** RMSE, Slope, and *R* ^2^ of Forces or Torques between the Model and QM Reference, Calculated for Fitting Each of the Two Phases to Their Separate Models and for Fitting Both Phases to the CHE_m_ Model

	Molecular Force	Molecular Torque	Atomic Force
Model	RMSD [kcal/(mol·Å)]	RMSD (kcal/mol)	RMSD [kcal/(mol·Å)]
CHE_n_/CHE_h_	1.14	1.97	7.69
CHE_m_	1.11	1.94	7.65

	Molecular Force	Molecular Torque	Atomic Force
Model		Slope	
CHE_n_/CHE_h_	0.922	0.874	0.959
CHE_m_	0.928	0.879	0.958

	Molecular Force	Molecular Torque	Atomic Force
Model		*R* ^2^	
CHE_n_/CHE_h_	0.925	0.893	0.955
CHE_m_	0.928	0.895	0.956

For a perfect fit, both the slope and *R* ^2^ are equal to one. It is clear from Table 1 that both fits, using separate models and the mixed-phase model, allow for accurate reproduction of the reference forces. The trend indicated by the slope and *R* ^2^ values is consistent with that observed for the RMSE.

It is worth pointing out that the atomic forces RMSE are substantially larger than the RMSEs of the net molecular force. This is expected as bonded forces are expected to be much stronger than intermolecular forces, especially for such a weakly interacting system. Interestingly, the CHE_m_ model also predicted the atomic force RMSE more accurately than the individual models, even though a single set of parameters was fitted to describe the bonded forces in two different environments. This is most likely a result of the CHE_m_ model using more atom types than the CHE_n_ model. For example, with two carbon types and two hydrogen types, cyclohexene will be described with five different bond terms in the CHE_n_ model. With the CHE_m_ model, a total of seven different bond terms are fit.

It is worth noting that the neat and hydrated phases were fitted with different reference methods. For the neat phase, which is dispersion-bound, DFT with an empirical dispersion is unlikely to provide the required accuracy. Consequently, MP2 was necessary for the neat phase model. However, we did not use MP2 for the hydrated system due to the requirement of AFM to include a large QM region with multiple hydration shells to adequately model a polar environment formed by water molecules. In the past, we have demonstrated that B3LYP-D3­(BJ) provides sufficient accuracy for predicting the HFE of small molecules.^35^ Using MP2 would be far more computationally expensive than using B3LYP.

Assuming that both MP2 and B3LYP provide the true intermolecular force, the exact method used to obtain such forces should not matter. In this sense, AFM provides a unique ability to model different types of interactions using different levels of theory, resorting to more costly QM methods only for systems where lower-cost alternatives are not expected to be sufficient. If the MP2 and B3LYP intramolecular potential energy surfaces are substantially different, then one would anticipate a drastic increase in atomic RMSE when one model is used to fit the intramolecular surface of two incompatible QM methods. The comparable atomic force RMSE between the mixed-phase CHE_m_ model and separate models indicates that MP2 and B3LYP provide very similar descriptions of cyclohexene. While the differences in dispersion are expected to affect the density,^43^ they do not prevent the creation of a high-quality mixed-phase model when two different electronic structure methods are fitted together.

### B Model Parameters and Details for Property Calculations

As mentioned previously, the cyclohexene models developed for simulations of the neat and hydrated phases will be denoted as CHE_n_ and CHE_h_, respectively. The model fitted for mixed-phase simulations will be referred to as CHE_m_. Although the parameters for both CHE_n_ and CHE_h_ have been reported previously, we provide a summary of the parameters for all three models in the Supporting Information. Gromacs 2019.6 was used when performing all simulations,^44^ and Gromacs input files for all three models are available online.^45^

Both the hydrated CHE_h_ and mixed-phase CHE_m_ models are designed to be compatible with the BLYPSP-4F water potential.^22^ The BLYPSP-4F model was previously developed with the AFM method by fitting against coupled-cluster quality forces obtained using the DFT-supplemental potential approach.^46^ It is a flexible four-site model with the negative charge located along the bisector of the HOH angle; it uses a Buckingham potential for short-range repulsion, along with a hydrogen bond term between the hydration site and the M site.^47^ Previous studies have shown that replacing the water model with an alternative one may not significantly impact solute properties, provided that the pair-specific solute–water interaction remains unchanged.^48^

For both the CHE_h_ and CHE_m_ models, the hydration free energy (*ΔG*_*hyd*_), the enthalpy of hydration (*ΔH*_*hyd*_), and the diffusion constant (*D*_*H*_) in water will be computed at 298 K. The details of these calculations will be provided in the Supporting Information.

For pure cyclohexene, we will determine various properties using both the CHE_n_ and CHE_m_ models. These properties include density (ρ), enthalpy of vaporization (*ΔH*_*vap*_), surface tension (γ), viscosity (η), diffusion constant (*D*_*P*_), isothermal compressibility (κ_*T*_), free energy of vaporization (*ΔG*_*vap*_), and boiling temperature (*T*_*b*_). Due to the higher computational demands, we will evaluate only the critical temperature (*T*_*C*_), pressure (*P*_*C*_), and density (ρ_*C*_) using the CHE_n_ model. The computational details for most of these properties are summarized in the Supporting Information, with key considerations highlighted below.

The γ was calculated using the following equation:^49^

1 $$\gamma =\frac{L_{Z}}{2}\left(P_{Z}-\frac{P_{X}+P_{Y}}{2}\right)$$

where *L*_*Z*_ is the length of the box in the *Z* dimension, and *P*_*X*_, *P*_*Y*_, and *P*_*Z*_ are the pressures along the *X*, *Y* and *Z* dimensions, respectively. This calculation was performed using a slab simulation consisting of 400 cyclohexene molecules, with a simulation box size of 4.1 × 4.1 × 12.0 nm. It is well established that precise determination of γ requires very long van der Waals cutoffs.^50^ Because our models use Buckingham potentials, they must be evaluated as tabulated potentials in Gromacs. Although Gromacs supports the use of Particle Mesh Ewald (PME)^51^ for dispersion, it is incompatible with tabulated potentials. As a result, we used a cutoff distance of 2.0 nm, which is the maximum possible value for the size of our simulation box. This may lead to a slight underestimation of the calculated surface tension due to the truncation of long-range van der Waals interactions.^50^

The critical properties of cyclohexene were determined using a simulation box with dimensions of 3.0 nm × 3.0 nm × 24.0 nm, containing 400 cyclohexene molecules. The simulations were performed at six different temperatures ranging from 360 to 460 K. At 360 K, the liquid slab was found to be approximately 8 nm thick. Due to the relatively small γ of cyclohexene, the liquid–vapor interface is highly rough and prone to forming large cavities that can penetrate deep into the liquid phase. To minimize the effect of these cavities on the liquid density measurement, we employed a relatively thick liquid slab in our simulations.

Due to fluctuations in the liquid density, simply selecting the region with the highest density to represent liquid density could lead to a systematic bias. To address this issue, we employ a two-step process to measure the density by first identifying a liquid region and then measure the density at the center of that region. The details of this density measurement process are provided in the Supporting Information.

Once the density and temperature have been determined, *T*_*C*_ will be obtained with the Wegner expansion^52^

2 $$\rho_{l}-\rho_{g}=A_{0}{\left|\tau \right|}^{\beta_{c}}+A_{1}{\left|\tau \right|}^{\beta_{c}+\Delta }+A_{2}{\left|\tau \right|}^{\beta_{c}+2\Delta }+A_{3}{\left|\tau \right|}^{\beta_{c}+3\Delta }$$

where τ = 1 – *T*/*T*_*C*_ and β_*c*_ and Δ are chosen to be 0.325 and 0.5, respectively, according to the 3D Ising universality class. To calculate ρ_*C*_, we fit the equation

3 $$\rho_{l}+\rho_{g}=2\rho_{C}+D_{1-\alpha \prime }{\left|\tau \right|}^{1-\alpha \prime }+D_{1}\left|\tau \right|$$

with *T*_*C*_ having been determined in the previous fit and an α′ value of 0.11 following previous work.^21^ To compute the *P*_*C*_, we used the pressure normal to the surface of the slab and fit Antoine’s equation,^53^

4 $$\ln\left(P\right)=A+\frac{B}{T+C}$$

via minimizing the least-squares error of ln­(*P*).

The *T*_*b*_ can also be determined by fitting the normal pressure to Antoine’s equation. The temperature at which the pressure reaches 1 atm is defined as *T*_*b*_. It is well established that the Antoine equation is not reliable over a wide range of temperatures. In practice, multiple Antoine equations are fitted for different temperature ranges.^54^ Therefore, we fit the Antoine equation from 380 to 460 K to determine *P*_*C*_, while *T*_*b*_ is measured by fitting the Antoine equation from 360 to 440 K and determining the temperature at which the vapor pressure is 1 bar. Additional details regarding the calculation of critical properties and *T*_*b*_ can be found in the Supporting Information.

Another method to compute *T*_*b*_ is by measuring the *ΔG*_*vap*_ and the density of the liquid, ρ_*l*_, at different temperature around *T*_*b*_. As *ΔG*_*vap*_ is defined as

5 $${\Delta G}_{vap}=-RT\;\ln\left(\frac{\rho_{g}}{\rho_{l}}\right)$$

the equilibrium vapor pressure can be computed from the vapor density, ρ_*l*_, by assuming the vapor exhibits the ideal gas behavior and

6 $$\ln\left(\frac{P_{g}}{P^{⊖}}\right)=\ln\left(\frac{RT\rho_{l}}{P^{⊖}}\right)-\frac{{\Delta G}_{vap}}{RT}$$

where the ideal gas law is used, and the standard state pressure *P* ^⊖^ is chosen as 1 atm.

To determine *T*_*b*_, *ΔG*_*vap*_ and ρ_*l*_ were computed at three different temperatures, 298.15, 344, and 364 K. The ln­(*P*_*g*_/*P* ^⊖^) is correlated against 1/*T* according to the Gibbs–Helmholtz equation.^55^ The correlations for the CHE_n_ and CHE_m_ models are shown in Figure 3. We refer to this method of determining *T*_*b*_ as the free energy method. And the determination of *T*_*b*_ through eq 4 was made by the Antoine method.

**Figure 3:**
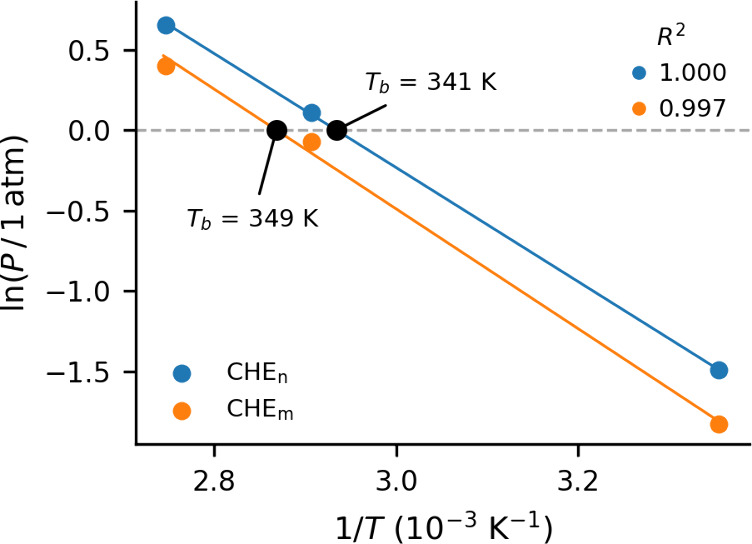
Boiling temperatures for the CHE_n_ and CHE_m_ models, obtained by correlating vapor pressure with inverse temperature according to the Gibbs–Helmholtz equation.

The solubility of liquid cyclohexene in water is quantified by the free energy of solvation, *ΔG*_*sol*_, which is computed as the sum of *ΔG*_*vap*_ and *ΔG*_*hyd*_. Specifically, *ΔG*_*vap*_ represents the free energy difference between the gas and liquid phases, while *ΔG*_*hyd*_ represents the free energy difference between the hydrated and the gas phases. By adding these two quantities, we obtain the free energy difference between the aqueous and liquid phases, *ΔG*_*sol*_. Notably, since solubility is a thermodynamic property that depends only on the free energy difference between two states, a mixed-state model is not required to compute this quantity.

As previously discussed, certain quantities require simulating bulk phases of cyclohexene and water such as γ_*int*_ between cyclohexene and water. The hydrated cyclohexene model, which is designed for infinite dilution, lacks parameters for cyclohexene–cyclohexene interactions and is therefore unsuitable for studying γ_*int*_. To calculate γ_*int*_, we employedthe CHE_*m*_ model in an orthorhombic simulation box containing 231 cyclohexene molecules and 1220 water molecules, with dimensions of 3.51 nm × 3.51 nm × 6.05 nm. Phase separation occurs along the *z*-axis, with the cyclohexene and water phases occupying approximately equal volumes within the simulation box. The van der Waals cutoff of 1.75 nm is used, which is the maximum allowed by the box. Further details regarding the simulation protocol for γ_*int*_ are provided in the Supporting Information.

## III Results and Discussion

All computed properties of cyclohexene are summarized in Table 2. With regard to the hydrated properties, both the CHE_h_ and CHE_m_ models predict *ΔH*_*hyd*_ in good agreement with experimental reference values, with deviations of approximately 0.6 kJ/mol or less. Additionally, the *D*_*H*_ values predicted by both models are consistent with each other, within the statistical uncertainty of our calculations. This is expected, as the CHE_h_ and CHE_m_ models have identical intermolecular parameters and differ only in intramolecular bonded terms, which are not expected to substantially affect either *ΔH*_*hyd*_ or *D*_*H*_. Unfortunately, we were unable to identify experimental values for *D*_*H*_, which limits its usefulness as a validation metric for the models.

**Table 2:** Experimental and Predicted Properties for Cyclohexene^a^

Property	Experimental Value	Predicted Value	
*ΔG*_*hyd*_	1.51 kJ/mol^56^	(H) 3.2 ± 0.3 kJ/mol	(M) 3.8 ± 0.3 kJ/mol
*ΔH*_*hyd*_	–32.20 kJ/mol^57^	(H) −31.6 ± 1.0 kJ/mol	(M) −31.8 ± 0.8 kJ/mol
*D*_*H*_	n/a	(H) (0.99 ± 0.01) × 10^–5^ cm^2^/s	(M) (0.99 ± 0.07) × 10^–5^ cm^2^/s
density	811. g/L^56^	(N) 839 ± 2 g/L	(M) 851 ± 1 g/L
*ΔH*_*vap*_	33.47 kJ/mol^56^	(N) 32.9 ± 0.1 kJ/mol	(M) 34.5 ± 0.3 kJ/mol
*ΔG*_*vap*_	18.911 kJ/mol^58^	(N) 17.4 ± 0.3 kJ/mol	(M) 18.2 ± 0.2 kJ/mol
*D*_*P*_	n/a	(N) (2.4 ± 0.2) × 10^–5^ cm^2^/s	(M) (1.9 ± 0.3) × 10^–5^ cm^2^/s
κ_*T*_	1.0366 × 10^–3^ MPa^–1^ ^59^	(N) (1.05 ± 0.05) × 10^–3^ MPa^–1^	(M) (1.0 ± 0.1) × 10^–3^ MPa^–1^
γ	26.17 mN/m^56^	(N) 22.6 ± 2.0 mN/m	(M) 25.4 ± 0.7 mN/m
η	0.625 mPa s^56^	(N) 0.49 ± 0.01 mPa s	(M) 0.66 ± 0.02 mPa s
*T*_*C*_	560.45 K^56^	(N) 513 K	
*P*_*C*_	44.3 bar,^56^ 49 bar^60^	(N) 45 bar	
ρ_*C*_	276.7 g/L,^60^ 283.25 g/L^56^	(N) 303.9 g/L	
*T*_*b*_	356.1 K^56^	(N) 344 K,^a^ 341 K^b^	(M) 349 K^b^
*ΔG*_*sol*_	20.42 kJ/mol,^c^ 19.6 kJ/mol,^d^ ^,^ ^56^	(H,N)^e^ 20.5 ± 0.4 kJ/mol	(M) 22.0 ± 0.4 kJ/mol
γ_*int*_	n/a		(M) 44.5 ± 0.7 mN/m

a (H) refers to the CHE_h_ model, (N) refers to the CHE_n_ model, and (M) refers to the CHE_m_ model.

b Computed with the Antoine method.

c Computed with the free energy method.

d Δ*G*_sol_ computed by summing the experimental Δ*G*_hyd_ and Δ*G*_vap_.

e Δ*G*_sol_ derived from the experimental solubility

f Both models were used to obtain this value. The error bar gives 68% confidence interval.

The *ΔG*_*hyd*_ values predicted by both models are in good agreement with the experimental reference,^56^ with a difference of approximately 2 kJ/mol, which is within the commonly accepted threshold for chemical accuracy. It is worth noting that the experimental *ΔG*_*hyd*_ is only 1.51 kJ/mol; a 2 kJ/mol absolute error would lead to a fairly larger percentage error, which does not indicate poor agreement. Although the CHE_m_ model yields slightly poorer ΔG_hyd_ values compared to those of the CHE_h_ model, the difference of 0.6 kJ/mol between the two models can be considered negligible.

For the pure phase properties, both the CHE_n_ and CHE_m_ models exhibit good agreement between their predictions and experimental values.^56–60^ The CHE_n_ model, which was specifically designed for the neat phase, overestimates the ρ by 3%, while the CHE_m_ model shows a slightly larger deviation of 5%. While the CHE_n_ model provides better agreement with experimental values^57,59^ for κ_*T*_ and *ΔH*_*vap*_, the CHE_m_ model shows improved agreement for the *ΔG*_*vap*_,^58^ as well as better predictions for the *γ, η* and *T*_*b*._ ^56^

We note that the difference between the CHE_n_ and CHE_m_ models is small for all of these properties. Since the models were fit to electronic structure methods rather than experimental data, there is no guarantee that the reference electronic structure methods will reproduce the exact experimental properties. Additionally, quantum nuclear effects are not considered in the computation of any of these properties. Therefore, although CHE_m_ seems to produce better agreement for a slightly larger number of properties, we do not have enough evidence to judge which model provides a better representation of the underlying electronic structure method used for training.

In contrast to the diffusion constant in water, *D*_*H*_, where the two models show good agreement with each other, the diffusion constant in pure phase *D*_*P*_ exhibits a significant difference of approximately 20% between the CHE_n_ and CHE_m_ models. The slightly higher cohesion energy in the CHE_m_ model leads to a substantially reduced *D*_*P*_. Applying the Stokes–Einstein equation^61^ with the predicted *D*_*P*_ and η yields estimated hydrodynamic radii of 1.85 Å for the CHE_n_ model and 1.74 Å for the CHE_m_ model. This corresponds to a substantial 6% difference in the hydrodynamic radius, which is surprisingly large considering the relatively small 2% difference in density between the two models.

Due to the higher computational cost of the liquid–vapor slab simulation, the critical properties were only computed for the neat cyclohexene model, CHE_n_. The parameters for the Wegner expansion are reported in Table 3, and the density–temperature diagram is shown in Figure 4. Compared with experiments, the critical temperature (*T*_*C*_) is 10% too low, and the critical density (ρ_*C*_) is 10% too high. It is important to note that the quality of the model may not be the sole factor contributing to these discrepancies. At the critical point, the correlation length diverges, and the finite box size of the simulation can significantly impact the extrapolation to criticality. Furthermore, the substantially lower γ of cyclohexene compared to liquid water created difficulties in determining liquid densities. As illustrated in Figure 5, large cavities will form near the liquid–vapor interface, and these cavities could diffuse into the slab. Although the cavity size decreased as they penetrated into the liquid, the low γ resulted in relatively long cavity lifetimes, leading to the persistence of smaller cavities in the central region, where liquid density measurements were taken. The presence of these cavities likely caused an underestimation of the liquid density and, consequently, hindered the accurate determination of *T*_*C*_.

**Figure 4:**
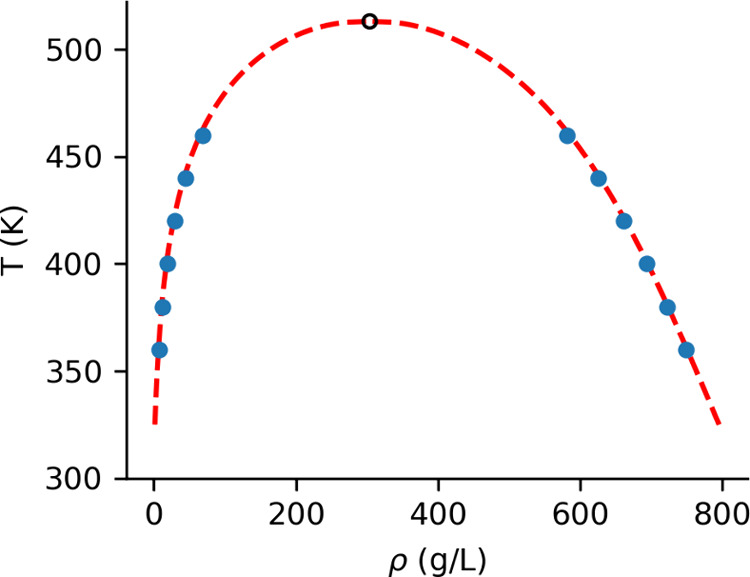
Phase diagram in the ρ*–T* plane. Solid circles represent the density measured in the simulations, the dashed line is the Wegner fit, and the hollow circle marks the critical point determined from the fit.

**Figure 5:**
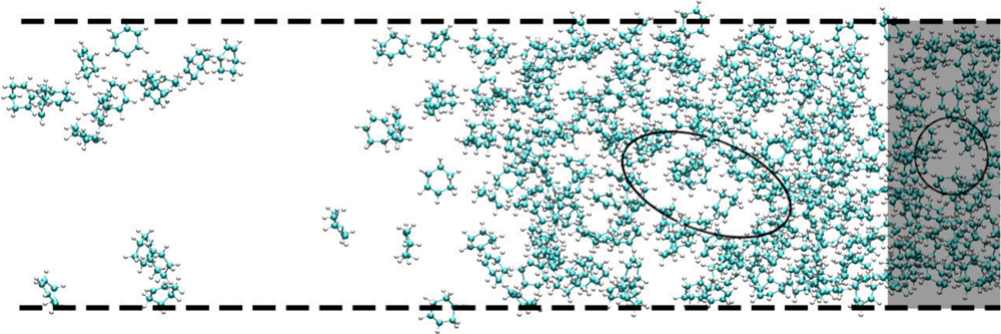
A snapshot of the slab at 460 K used for density measurements. Cavities formed near the liquid–vapor interface (large oval) penetrate deep into the slab;smaller cavities (small oval) can still be observed in the shaded region, where the liquid density is measured.

**Table 3:** Parameters of the Wegner Expansion Obtained by Fitting the Liquid and Vapor Phase Densities of the CHE_n_ Model

*A*_0_ (kg/L)	*A*_1_ (kg/L)	*A*_2_ (kg/L)	*A*_3_ (kg/L)	*D*_1−α′_ (kg/L)	*D*_1_ (kg/L)
579.5	4637.6	–8973.0	5706.9	–766.1	1496,8

The critical pressure (*P*_*C*_) and boiling temperature (*T*_*b*_) were determined from the high-temperature and low-temperature Antoine fits of the normal pressure of the slab (Figure 6). As the Antoine equation may not be sufficiently accurate when fitted to the full temperature range, it is common practice to fit multiple Antoine equations as the temperature varies. The parameters of the high temperature fit used for obtaining *P*_*C*_ and the low temperature fit for obtaining *T*_*b*_ are shown in Table 4. While the actual Antoine’s parameters differ substantially, it is clear from Figure 6 that the high- and low-temperature fits agree well in the region where they overlap.

**Figure 6:**
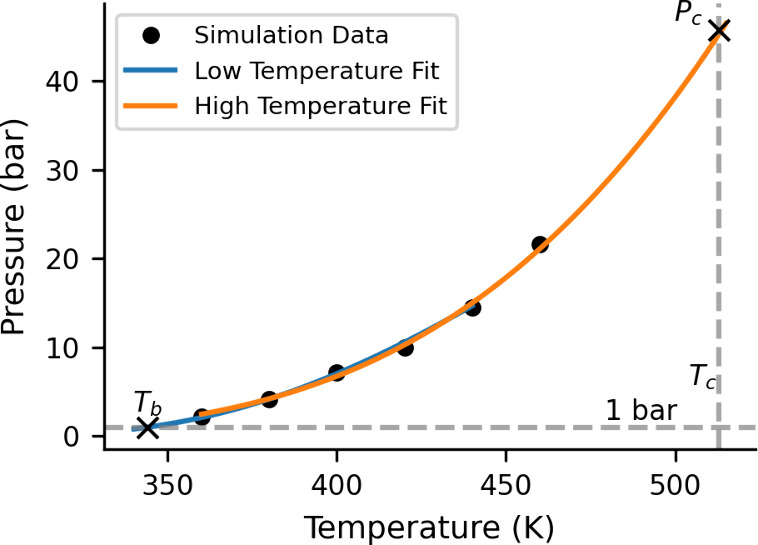
Pressure and temperature diagram for the CHE_n_ model. The slab normal pressure measured from the simulation (dots) was fitted to the two separate Antoine questions: one for the high-temperature range (orange) and one for the low-temperature range (blue). The two fits agree well for intermediate temperatures falling within both fitting ranges.

**Table 4:** Fitted Parameters for the Antoine Equation over the Selected Temperature Ranges for the CHE_n_ Model

*T* range (K)	*A*	*B* (K)	*C* (K)
360–440	5.7	–612.2	–236.3
380–460	9.8	–2186.0	–43.7

The *P*_*C*_ agrees well with one of the two experimental measurements. However, it is worth noting that *P*_*C*_ is measured at the model *T*_*C*_, which is 10% too low.

The *T*_*b*_ of the CHE_n_ model computed with the Antoine method is 341 K, which agrees within 1% of that obtained with the free energy method. This indicates high consistency between these two methods. While the free energy method of computing *T*_*b*_ is fairly straightforward, we are not aware of its use in the literature. The good consistency between the more traditional but expensive Antoine method and the free energy method suggests that the latter is an effective tool for the computational determination of *T*_*b*_.

The *ΔG*_*sol*_ estimated by combining the CHE_n_ and CHE_h_ models is almost identical to the experimental reference.^56,57^ The *ΔG*_*sol*_ estimated using the CHE_m_ model is also in very good agreement, demonstrating the ability of the AFM models to predict solubility. This is expected, as the models give good predictions of both *ΔG*_*vap*_ and *ΔG*_*hyd*._

The interfacial tension γ_int_ between cyclohexene and water can be computed only with the two phase CHE_m_ model, which gives a γ_*int*_ of 44.5 mN/m. We were unable to obtain a reliable experimental γ_*int*_ from the literature. However, the γ_*int*_ values of cyclohexane and benzene with water are both known,^62^ with the former being 50 mN/m and the latter being 34.10 mN/m. Our prediction appears to be closer to that of cyclohexane, which is more similar to that of cyclohexene than benzene is. The double bond in cyclohexene is expected to increase the polarizability of the molecule, which is expected to strengthen the interaction of the molecule with water and reduce the γ_*int*_. This suggests that it is highly plausible that γ_*int*_ of the CHE_m_ model is close to the true value.

## IV Summary and Conclusion

Despite the significant advances made by electronic structure methods in predicting molecular-scale energetics, the prediction of macroscopic properties at finite temperatures using these methods has long been considered a major challenge. In this study, we demonstrate a novel approach to obtaining a wide range of macroscopic properties for a molecule using only electronic structure calculations. As a proof-of-concept, we selected cyclohexene as the target molecule. The neat phase properties were determined by fitting MP2/def2-TZVP^63^ quality electronic structure calculations to an AFM model, denoted as CHE_n_. Similarly, the hydrated phase properties were obtained by fitting B3LYP-D3­(BJ) calculations to another AFM model, CHE_h_. Using these two models, we successfully predicted 15 distinct properties that require extensive ensemble averaging. Where experimental reference values are available, our AFM-based model predictions exhibit excellent agreement with the experimental data.

While using separate models for hydrated and neat phases allows for the determination of solubility, we also developed a mixed-phase model, CHE_m_, by fitting to both phases simultaneously. By using partial charges also in the neat phase and using more atom types, the CHE_m_ model yields slightly better RMSE of the fits when compared to fitting each individual phase with its own separate model. Clearly, if a neat model were created with partial charges and the same atom types as the CHE_m_ model, fitting one model to each phase would necessarily produce smaller RMSE’s. The performance penalty incurred by fitting the two phases together is no greater than the penalty associated with using a less complex single phase model lacking partial charges.

This mixed-phase model enables the investigation of systems where liquid cyclohexene and water coexist, allowing us to determine the γ_int_ between the two phases. The predicted γ_int_ is between the known experimental values for cyclohexane and benzene, but closer to that of cyclohexane, which is consistent with the value expected based on the molecule’s similarity to the two molecules.

While B3LYP-D3­(BJ) has been shown to provide good accuracy for fitting hydrated models, a more rigorous treatment of dispersion may be important for weakly bound systems, such as neat cyclohexene. AFM offers the unique ability to fit cyclohexene–cyclohexene interactions to MP2 with SAPT dispersion and cyclohexene–water interactions to B3LYP-D3­(BJ), thereby allowing the more expensive post-Hartree–Fock approach to be used only when it is required.

Although experimental reference values are not available for some properties, AFM models successfully predicted all of the properties, for which the experimental reference values are known. Even for *T*_*c*_ and ρ_*c*_, a 10% deviation is not poor agreement, especially considering the experimentally determined reference *P*_*c*_ values reported by two sources differ by 10%.^56,60^ It is clear that AFM models are valuable for predicting properties that are not available experimentally.

As the sole objective of AFM is to reproduce electronic structure forces in the condensed phase for conformations that are important for the thermodynamic condition of interest, the success of the AFM models ultimately reflects the accuracy of the underlying electronic structure methods. No experimental data were fitted during the development of these models. AFM extends the time and length scales of the underlying electronic structure simulations, thereby enabling the computation of the finite-temperature properties of an ensemble of molecules. A key difference between AFM models and traditional force fields is the elimination of combining rules, which we believe is a crucial factor in the accuracy of the AFM model.

The ability to predict macroscopic properties solely based on electronic structure calculations through AFM represents a significant new capacity in computational materials science, particularly for novel molecules where experimental data may be scarce or nonexistent. This capability greatly enhances our ability to explore new chemicals at substantially reduced cost and increased speed, making it a major advance in the application of electronic structure theory.

## Supplementary Material



## Notes

Cyclohexene model development protocol, parameters for the models, and detailed methodology for property calculations (PDF)
