# Supplementary material for: Predicting Properties of Cyclohexene with Electronic Structure Methods through Adaptive Force Matching
Source: J Phys Chem C Nanomater Interfaces. 2026 May 5;130(20):7100–8. doi: 10.1021/acs.jpcc.6c00465 (PMC13200184; doi:10.1021/acs.jpcc.6c00465)
Supplement: Supplementary file 1 [file jp6c00465_si_001.pdf]

Supplementary Information (SI)

Predicting Properties of Cyclohexene with Electronic Structure Methods through Adaptive Force Matching.

Raymond Weldon[a], Feng Wang[a]*

[a]Department of Chemistry and Biochemistry,

University of Arkansas,

Fayetteville,

AR 72701, USA

*Email: fengwang@uark.edu

1. Cyclohexene Model Development Protocol

The neat cyclohexene model is CHE_n and the hydrated cyclohexene model is CHE_h. Detailed procedures for the development of the both models have been reported in a prior publication.[1] A brief description of the procedure for developing these models, along with a description for the fitting of the mixed phase CHE_m model, is provided below.

For both the CHE_n and CHE_h models, the dispersion was modeled using the short-range damped (SRD) dispersion

$$\mathrm{SRD(r)} = \frac{C_6}{r^6+r_0^6} + \frac{C_8}{r^8+r_0^8} \tag{1}$$

For the CHE_n model, the parameters for the SRD were determined by fitting the E2 dispersion energies[2] from Symmetry Adapted Perturbation Theory (SAPT) using the def2-TZVPD basis set.[3-4] When fitting dispersion parameters, numerical stability issues can sometimes cause one of the C_6 or C_8 parameters to be positive. In those cases, we always remove the C_8 parameter first and refit. The final C_6 and C_8 parameters are required to be negative.

In each AFM iteration, the MD step for the fitting of the CHE_n model comprises two MD simulations at 1 bar with temperatures of 298 K and 328 K. The reference forces for the QM/MM step were computed with the MP2 method using the def2-TZVPD basis set without using a MM region as the molecule is not polar. No partial charges are used on any of the atoms during the fitting in the FM step. The only intermolecular interaction is the short-range repulsion term modeled with an exponential form:

$$\mathrm{EXP(r)} = Ae^{-\alpha r} \tag{3}$$

For the CHE_n model, the bonded interactions are modelled with the harmonic bond and angle terms and the dihedral interactions are modeled using the equation.

$$E_{dih}(\omega) = V_D[1 + \cos(m * \theta - \delta)] \tag{4}$$

A total of eight iterations of AFM were performed with the final four generations fitted together to obtain the final force field parameters reported in Table 1.

As the CHE_h model was fit against B3LYP-D3(BJ) forces, the dispersion parameters for this model were fitted against Grimme's D3 dispersion with Beck-Johnson damping.[5-6] As our prior work suggests that dispersion is less important for hydrated systems,[7] the $1/r^8$ contribution to dispersion is ignored for the CHE_h model by setting C_8 in Eq. 1 to zero.

During AFM iterations, MD simulations were performed at 1 bar with temperatures set at 298 K and 328 K. The QM reference method for the QM/MM calculations was B3LYP-D3(BJ). A mixed basis set is used where heavy atoms are described using aug-cc-pVTZ,[8-9] and the hydrogen atoms are modeled using the cc-pVTZ basis set. The center zone of the QM region includes cyclohexene and 5 QM water molecules; the center zone is surrounded by the boundary zone, which is also modeled with QM. Water molecules with in 9 Å of cyclohexene are included in the MM region. Partial charges are used on cyclohexene atoms, with repulsion modeled using the EXP form shown in Eq. 2. The bonded terms have the same forms as the CHE_n model, although the parameters are fitted independently. A total of eight generations of AFM were run, with the last five generations used for the global fit. The parameters for this model are summarized in Table 2.

As discussed in the main text, the mixed phase model, CHE_m can be used to describe liquid cyclohexene in contact with liquid water. This model was fit by constraining the partial charges to those of the CHE_h model. However, the global training sets for both the CHE_n and CHE_h models were fitted together without performing any new QM or QM/MM calculations. Only one set of bonded parameters is used regardless of whether the cyclohexene is in hydrated or neat environments. The dispersion parameters of the CHE_n and CHE_h models are used for the CHE_m without refitting. The parameters for the CHE_m model are summarized in Table 3.

2. Parameters for the CHE_n, CHE_h and CHE_m models.

Table S1: Neat Cyclohexene (CHE_n) Parameters

Nonbonded

At1	At2	Interaction	A_{ij} (kcal/mol)	α ($Å^{-1}$)	Interaction	C_6 (kcal $Å^6$/mol)	r_0 (Å)	C_8 (kcal $Å^8$/mol)	r_0 (Å)
H1	H1	EXP	2122.32	4.038					
H0	H1	EXP	1918.45	3.964					
H0	H0	EXP	991.334	3.492					
C1	H1	EXP	61733.69	4.889					
C1	H0	EXP	9776.646	3.926					
C0	H0	EXP	157594.11	5.428					
C1	C1	EXP	61288.735	3.399	SRD	-951.911	2.121		
C0	C1	EXP	709963.2	4.186	SRD	-955.114	2.083		
C0	C0	EXP	123751.19	3.351	SRD	-678.539	2.045	-25634.408	2.045

Charges

Atom	Charge (e)
C0	0
C1	0
H0	0
H1	0

Bonds

At1	At2	r_e (Å)	k_b (kcal/mol $Å^2$)
C1	C1	1.3280929	1219.0509
C1	C0	1.4744487	569.88653
C0	C0	1.5139261	531.97035
C0	H0	1.0967954	727.26706
C1	H1	1.0896179	768.35959

Angles

At1	At2	At3	ϑ_e (degrees)	k_ϑ (kcal/mol rad^2)
C0	C0	C0	108.73029	119.39651
H0	C0	C0	107.64887	90.909261
H0	C0	H0	104.45308	70.87463
C0	C0	C1	105.42594	108.11698
H0	C0	C1	106.84149	85.20418
C0	C1	C1	124.44618	82.648246
H1	C1	C0	126.10906	62.105783
H1	C1	C1	127.10789	63.712474

Dihedrals

At1	At2	At3	At4	V_D (kcal/mol)	m	δ (degree)
C0	C0	C0	C0	0.88348603	3	0

C0	C0	C0	C1	0.34881548	3	0
C0	C0	C1	C1	-0.97606949	3	0
C0	C1	C1	C0	4.4580735	2	180
H1	C1	C1	C0	7.4973906	2	180
H1	C1	C1	H1	3.6177467	2	180

Table S2: Hydrated Cyclohexene (CHE_h) parameters

Nonbonded

At1	At2	Interaction	A_{ij} (kcal/mol)	α (Å^{-1})	Interaction	C_6 (kcal Å^6/mol)	r_0 (Å)
C1	HW	EXP	1648.381	3.421			
C3	HW	EXP	2912.838	3.211			
C1	C3	EXP	13430.342	3.6			
C2	C2	EXP	51702.419	3.6			
C1	H2	EXP	55385.867	3.6			
C1	H3	EXP	5110.1456	3.6			
C3	H1	EXP	453843.17	3.6			
C3	H2	EXP	1946.6996	3.6			
H3	OW	EXP	6012.78	3.635			
H2	OW	EXP	4990.035	3.609			
H1	OW	EXP	4999.225	3.615			
C3	OW	EXP	686056.61	4.422	SRD	-1005.293	1.997
C2	OW	EXP	148215.73	3.699	SRD	-1247.181	1.997
C1	OW	EXP	173473.58	3.898	SRD	-637.39	2.035

Charges

Atom	Charge (e)
C1	-0.33379
C2	0.15181
C3	-0.18029
H1	0.15577
H2	0.03635
H3	0.0669

Bonds

At1	At2	r_e (Å)	k_b (kcal/mol Å^2)
C3	C3	1.5207056	473.03372
C3	C2	1.5186424	462.85191
C3	H3	1.0942393	698.67169
C2	H2	1.09473	686.28384
C2	C1	1.4854902	523.19254
C1	C1	1.3243816	1131.9171
C1	H1	1.0764063	739.89916

Angles

At1	At2	At3	ϑ_e (degrees)	k_ϑ (kcal/mol rad^2)
C2	C3	C3	111.04378	139.1194
C2	C3	H3	108.14951	92.578554
C3	C3	H3	107.95417	90.7298
C3	C2	H2	106.10407	92.742684
H3	C3	H3	104.98906	72.382269
H2	C2	H2	100.71389	70.697908

C3	C2	C1	110.27912	130.45271
C1	C2	H2	104.26539	89.085351
C2	C1	C1	137.58736	112.3769
C2	C1	H1	138.135	57.320015
C1	C1	H1	138.73898	63.533142

Dihedrals

At1	At2	At3	At4	V_D (kcal/mol)	m	δ (degree)
C3	C2	C1	C1	-0.28732368	3	0
C3	C3	C2	C1	0.35801426	3	0
C2	C1	C1	C2	1.9683354	2	180
C2	C3	C3	C2	0.53557512	3	0
C2	C1	C1	H1	6.3766554	2	180

Table S3: Mixed phase Cyclohexene (CHE_m) Parameters

At1	At2	Interaction	A_{ij} (kcal/mol)	α (Å^{-1})	Interaction	C_6 (kcal Å^6/mol)	r_0 (Å)	C_8 (kcal Å^8/mol)	r_0 (Å)
C1	HW	EXP	1648.381	3.421					
C3	HW	EXP	2912.838	3.211					
H1	H1	EXP	2460.473	4.304					
H1	H2	EXP	2732.5	4.259					
H1	H3	EXP	2052.177	4.116					
H2	H2	EXP	1372.478	3.672					
H2	H3	EXP	1086.789	3.54					
H3	H3	EXP	752.629	3.341					
C1	C1	EXP	191785.81	3.892	SRD	-951.911	2.121		
C1	C2	EXP	836868.11	4.216	SRD	-955.114	2.083		
C1	C3	EXP	479792.73	4.133	SRD	-955.114	2.083		
C2	C2	EXP	558257.61	3.816	SRD	-678.539	2.045	-25634.408	2.045
C2	C3	EXP	187783.35	3.46	SRD	-678.539	2.045	-25634.408	2.045
C3	C3	EXP	167668.24	3.438	SRD	-678.539	2.045	-25634.408	2.045
C1	H1	EXP	10462.383	3.852					
C1	H2	EXP	3219.061	3.356					
C1	H3	EXP	4654.91	3.448					
C3	H1	EXP	929856.6	6.04					
C3	H2	EXP	226536.06	5.5					
C3	H3	EXP	90337.704	5.021					
H3	OW	EXP	6012.78	3.635					
H2	OW	EXP	4990.035	3.609					
H1	OW	EXP	4999.225	3.615					
C3	OW	EXP	686056.61	4.422	SRD	-1005.293	1.997		
C2	OW	EXP	148215.73	3.699	SRD	-1247.181	1.997		
C1	OW	EXP	173473.58	3.898	SRD	-637.39	2.035		

Atom	Charge (e)
C1	-0.33379
C2	0.15181
C3	-0.18029
H1	0.15577
H2	0.03635
H3	0.0669

Bonds

At1	At2	r_e (nm)	k_b (kcal/mol Å^2)
C1	C1	1.32318	1218.252
C1	C2	1.47283	559.416
C1	H1	1.08731	766.193
C2	C3	1.51894	541.407
C2	H2	1.09770	719.978

C3	C3	1.51119	488.975
C3	H3	1.09530	726.701

Angles

At1	At2	At3	ϑ_e (degrees)	k_ϑ (kcal/mol rad^2)
C1	C1	C2	120.03522	85.5447
C1	C1	H1	126.32883	63.2042
C2	C1	H1	126.25086	61.4635
C1	C2	C3	105.26575	107.0021
C1	C2	H2	105.61129	88.4278
C3	C2	H2	106.52477	92.2050
H2	C2	H2	101.69868	71.2039
C2	C3	C3	110.08278	122.1943
C2	C3	H3	108.22564	90.1331
C3	C3	H3	107.51094	89.5468
H3	C3	H3	105.71637	72.2137

Dihedrals

At1	At2	At3	At4	V_D (kcal/mol)	m	δ (degree)
C2	C1	C1	C2	5.499278203	2	180
C2	C1	C1	H1	7.37893499	2	180
C1	C1	C2	C3	-0.84475925	3	0
H1	C1	C1	H1	3.70555521	2	180
C1	C2	C3	C3	0.396779207	3	0
C2	C3	C3	C2	0.896064866	3	0

2. Details for the Methodology of the Property Calculations.

(a) Properties in the aqueous solution

The water model for the computation of cyclohexene hydration free energy (ΔG_{hyd}), the enthalpy of hydration (ΔH_{hyd}), and the diffusion constant (D_H) is BLYPSP-4F. Long-range electrostatics were described using the particle mesh ewald (PME) method[10] with a B-spline order of 4 and a reciprocal space spacing of 0.12 nm. The real space cut off is 1.0 nm and a real space tolerance of 10^{-5} is used. The van der Waals cutoff is also 1.0 nm. The simulation time step size was chosen to be 0.5 fs. All constant pressure simulations were performed at 1 bar with the Parrinello-Rahman barostat[11-12] using a 5 ps relaxation constant. All simulation were performed at 298 K. All simulations were performed with Gromacs version 2019.6.

The hydration free energy (ΔG_{hyd}) is computed using the Bennet Acceptance Ratio (BAR) method as implemented in Gromacs. The BAR simulation was performed both in the aqueous solution and in the gas phase with the Gromacs keyword "couple-intramol" set to yes, and the ΔG_{hyd} is computed as the difference between the alchemical free energy in the solution phase and the gas phase.

The solution phase simulations were performed under NPT conditions with one cyclohexene in 343 water molecules. The gas phase simulations were performed under NVT conditions using a cubic box that is $(3\ nm)^3$ containing one cyclohexene molecule. The temperature coupling for both the aqueous and gas phase simulation was enforced with the Langevin thermostat[13] with an inverse friction coefficient of 2 ps^{-1}.

A total of 30 windows are used for the BAR simulations in each phase. The Coulombic interaction is switched off in 10 windows, followed by switching off van der Waals interactions in another 20 windows. Each trajectory of the BAR simulation was run for 5 ns for the solution phase simulation but 11 ns for the gas phase simulation. The statistics for the alchemical free energy calculation were collected after discarding the first nanosecond of the solution and gas phase simulations in each window.

A soft-core potential[14] was used to improve sampling of the short-range repulsion. The soft-core potential was configured with the sc-alpha parameter set to 1. With tabulated potentials, the 2019.6 version of Gromacs computes the soft-core radius using C12 and C6 for each pair of atoms in the topology file following the formula

$$r_{sc} = \left(\frac{\mathrm{C12}}{\mathrm{C6}}\right)^{\frac{1}{6}} \tag{5}$$

As the C6 parameter should not be adjusted to allow long-range dispersion correction to energy and pressure to be properly computed, the control of r_{sc} was accomplished by adjusting C12 and scaling the corresponding column in the table file. The C12 is set to make r_{sc} to be 0.265 nm.

The enthalpy of hydration ($\Delta\mathrm{H}_{\mathrm{hyd}}$) was computed with the following equation

$$\Delta\mathrm{H}_{\mathrm{hyd}} = \langle\mathrm{U}\rangle_{\mathrm{solution}} - (\langle\mathrm{U}\rangle_{\mathrm{water}} + \langle\mathrm{U}\rangle_{\mathrm{solute}}) + \mathrm{PV}_{\mathrm{solution}} - \mathrm{PV}_{\mathrm{water}} - \mathrm{RT} \tag{6}$$

where $\langle\mathrm{U}\rangle_{\mathrm{solution}}$ and $\langle\mathrm{U}\rangle_{\mathrm{water}}$ were the average configuration energies of the solution and a neat water box, respectively. $\langle\mathrm{U}\rangle_{\mathrm{solute}}$ is the intramolecular solute energy obtained by simulating one cyclohexene molecule in a cubic box of $(3\ \mathrm{nm})^3$ with Coulombic interactions between periodic images cut off. The temperature of the single cyclohexene simulation is controlled with a Langevin thermostat with an inverse friction coefficient of 2 ps^{-1}, and the temperature of the liquid phase simulations was controlled with the Nose-Hoover[15-16] thermostat with a relaxation constant of 2 ps. The volumes, $V_{solution}$ and V_{water} are average box volumes of the solution and neat water boxes, respectively. All statistics were obtained from 200 ns MD simulations.

The diffusion constant of cyclohexene in water (D_H) was measured with a cubic box containing one solute and 343 water molecules. A total of 20 NVT simulations were run for 2.4 ns each, and the D_H was measured using the Einstein Equation from the last 2 ns of trajectory, giving a total of 40 ns worth of statistics. The box sizes of the NVT simulations were determined with a separate NPT simulation, averaging 4 ns of a 5 ns trajectory. The 20 initial conformations were selected from the last 1 ns of the NPT simulation. A Nose-Hoover thermostat relaxation constant of 5 ps was used during the NVT simulations.

(b) Properties of Neat Cyclohexene

A series of properties for the neat cyclohexene was computed. Unless noted otherwise, all neat phase simulations were performed with a 1 fs timestep. To enable this relatively large timestep, the hydrogen mass was set to that of the tritium. Unless otherwise specified, temperature control was achieved with the Nose-Hoover thermostat with a relaxation time of 2 ps, and pressure control was achieved with a Parrinello-Rahman barostat with a relaxation time of 5 ps. The van der Waals cutoff was set to 1.3 nm except for the determination of surface tension, γ, the interfacial surface tension, γ_{Int}, and the boiling and critical point simulations. For models including partial charges, electrostatic interactions were handled with the PME method, with the real space cutoff matching the van der Waals cutoff for each simulation. The B-spline order was 4 and the reciprocal space spacing was 0.12 nm, with a real space tolerance of 10^{-5}. The long-range correction to energy and stress due to van der Waals was enabled in Gromacs. The neat cyclohexene model includes a $1/r^8$ dispersion term, which Gromacs ignores when computing long-range correction to pressure and energy. To compensate for this, for all NPT simulations, a long-range correction to pressure due to the $1/r^8$ dispersion was computed. [17] This correction is added to the target pressure of the barostat in the Gromacs mdp file to ensure the final pressure, considering the long range correction due to $1/r^8$ dispersion, is correct. Because the long-range correction depends on density, a density estimate obtained by a 3 ns NPT simulation was used when computing such corrections.

The density ρ was computed at 293 K and 1 atm using a cubic box containing 125 cyclohexene. The density was measured over the final 20 ns of a 25 ns NPT simulation. Due to the use of a heavy isotope of hydrogen during the MD simulations, all reported mass densities were obtained by converting the mass density from the simulation to number density and back to mass density using the mass of the elements.

The enthalpy of vaporization, ΔH_{vap}, at 298 K and 1 atm was computed using equation (7)

$$\Delta \mathrm{H}_{\mathrm{vap}} = \langle \mathrm{V}_{\mathrm{gas}} \rangle - \langle \mathrm{V}_{\mathrm{liq}} \rangle + \mathrm{RT} \tag{7}$$

where $\langle \mathrm{V}_{\mathrm{gas}} \rangle$ and $\langle \mathrm{V}_{\mathrm{liq}} \rangle$ are average potential energies of the gas and liquid phases, respectively. $\langle \mathrm{V}_{\mathrm{gas}} \rangle$ were obtained by simulating a single cyclohexene molecule in a cubic box of $(3\ \mathrm{nm})^3$ for 110 ns, with the average taken over the last 100 ns. The temperature for the gas phase simulation was controlled with a Langevin thermostat with an inverse friction

coefficient of 2 ps^{-1}. $\langle V_{liq} \rangle$ was computed using a cubic box containing 125 molecules, averaging over the final 20 ns of a 25 ns simulation. The isothermal compressibility, κ_T, was computed using the volume variance of the final 20 ns of the liquid simulation used for determining ΔH_{vap} using the fluctuation-dissipation theorem.

The viscosity η and diffusion constant D_P were measured at 298 K using a cubic box containing 165 molecules. NVT simulations were performed using a 0.5 fs timestep, with the temperature maintained with a Nose-Hoover thermostat and a relaxation constant of 5 ps. The isotope mass of ^{1}H is used for these simulations. The box volume was determined by averaging over the final 4 ns of a 5 ns NPT simulation at 1 bar. η was computed as the long time limit of the sheer viscosity using method of Zhang et al.[18] 100 NVT simulations were performed for 5 ns each, with the off-diagonal elements of the stress tensor saved once every 5 fs. While the trajectories were simulated with Gromacs 2019.6, the η as a function of time was obtained using the Green Kubo relationship[19] with the 2024.5 version of Gromacs. The time dependent viscosity was fitted to the double exponential form with the weight following the power law function as recommended by Zhang and the t_{cut} is chose to be the time the RMSD drop to less than 40% of the viscosity. D_p was computed by computing the mean square displacement using the final 2 ns of a 2.4 ns NVT simulation, and the fit was performed in the range from 5 to 15 ps.

Surface tension γ at 298 K was measured using a slab containing 400 cyclohexene molecules. The orthorhombic box is 4.1 nm × 4.1 nm × 12.00 nm, which allows the use of a 2.0 nm van der Waals cutoff. The distance between the two surfaces of the slab is approximately 8 nm at 298 K. γ was measured using the following equation[20]

$$\gamma = \frac{L_Z}{2}\left(P_Z - \frac{P_X + P_Y}{2}\right) \tag{8}$$

where L_Z is the length of the box in the Z-dimension, and P_X, P_Y, and P_Z are the pressures in the X, Y and Z dimensions, respectively. The γ was determined from the final 50 ns of a 52 ns simulation.

The boiling temperature T_b and the critical properties, T_C, P_C, and ρ_C were computed using a 400 cyclohexene box that is 3 nm x 3 nm x 24 nm. The slab simulations were performed between 360 K and 460 K in 20 K increments. At 360 K, the liquid is approximately 8 nm thick. After a 2 ns equilibration, each temperature is simulated for 540 ns for density and pressure measurements. The liquid and gas densities were measured using a protocol reported by Hu and

Wang.[21] The average z-density across a 7 nm window is computed. The window that exhibits the largest average density is identified to contain the liquid slab. To minimize the influence of the liquid-vapor interface, the liquid density is determined as the density across the 0.5 nm region at the center of the window. The gas density is taken as the density in a 0.5 nm window 12 nm away from the center of the liquid. In order to avoid center of mass drift, the density measurement is performed after dividing each trajectory into 50 ps chunks. The reported mass densities reflect those of cyclohexene without tritium substitution.

The T_C was determined by Wegner expansion[22]

$$\rho_l - \rho_g = A_0|\tau|^{\beta_c} + A_1|\tau|^{\beta_c+\Delta} + A_2|\tau|^{\beta_c+2\Delta} \tag{9}$$

where $\tau = 1 - T/T_C$, and β_c and Δ are chosen to be 0.325 and 0.5, respectively, according to the 3D Ising universality class. After T_C is determined, ρ_C is obtained by fitting the equation

$$\rho_l + \rho_g = 2\rho_C + D_{1-\alpha'}|\tau|^{1-\alpha'} + D_1|\tau| \tag{10}$$

with an α' value of 0.11.[23]

P_C and T_b were determined by fitting the normal pressure of the slab with the Antoine's equation in log form

$$\ln(P) = A + \frac{B}{T+C} \tag{11}$$

For computing T_b, Eq. 11 was fit in the range from 360 K to 440 K, whereas the range from 380 K to 460 K was used for computing P_C. P_C was computed as the pressure at T_C, and T_b was computed as the pressure at 1 bar.

(c) Determination of interface tension γ_{Int}

The water-cyclohexene interfacial tension, γ_{Int}, was computed using an orthorhombic box that is 3.51 nm x 3.51 nm x 6.0525 nm under 294 K. This box contains 231 cyclohexene molecules and 1220 water molecules, occupying approximately equal volume. The 6.0525 nm was obtained by adjusting the z-dimension manually until the pressure normal to the slab from a 1 ns simulation is within ± 15 bar of a 0 bar target pressure. The γ_{Int} was obtained from the

final 50 ns of a 52 ns trajectory using a van der Waals cutoff of 1.75 nm. The average normal pressure of the 50 ns production simulation is 1.76 bar.

(d) Determination of error bars

The error bars for D_P, ΔG_{hyd}, and ΔG_{vap} were reported by Gromacs. The error bar on ΔG_{sol} was computed with the error propagation formula using the error bars of ΔG_{hyd}, and ΔG_{vap}. All other error bars were determined as the standard error of the mean by dividing the data into multiple sets.

References:

1. Weldon, R.; Wang, F., Exploring the promise and limitations of point-charge-free potentials for hydrocarbon modeling. *Scientific Reports* **2025,** *15*, 23055.
2. Szalewicz, K., Symmetry-adapted perturbation theory of intermolecular forces. *WIREs Computational Molecular Science* **2012,** *2*, 254-272.
3. Weigend, F.; Ahlrichs, R., Balanced basis sets of split valence, triple zeta valence and quadruple zeta valence quality for H to Rn: Design and assessment of accuracy. *Physical Chemistry Chemical Physics* **2005,** *7*, 3297-3305.
4. Rappoport, D.; Furche, F., Property-optimized Gaussian basis sets for molecular response calculations. *The Journal of Chemical Physics* **2010,** *133*, 134105.
5. Becke, A. D.; Johnson, E. R., A density-functional model of the dispersion interaction. *The Journal of Chemical Physics* **2005,** *123*, 154101.
6. Grimme, S.; Ehrlich, S.; Goerigk, L., Effect of the damping function in dispersion corrected density functional theory. *Journal of Computational Chemistry* **2011,** *32*, 1456-1465.
7. Zheng, D.; Wang, F., Performing Molecular Dynamics Simulations and Computing Hydration Free Energies on the B3LYP-D3(BJ) Potential Energy Surface with Adaptive Force Matching: A Benchmark Study with Seven Alcohols and One Amine. *ACS Physical Chemistry Au* **2021,** *1*, 14-24.
8. Dunning, T. H., Jr., Gaussian basis sets for use in correlated molecular calculations. I. The atoms boron through neon and hydrogen. *The Journal of Chemical Physics* **1989,** *90*, 1007-1023.
9. Kendall, R. A.; Dunning, T. H., Jr.; Harrison, R. J., Electron affinities of the first-row atoms revisited. Systematic basis sets and wave functions. *The Journal of Chemical Physics* **1992,** *96*, 6796-6806.
10. Darden, T.; York, D.; Pedersen, L., Particle mesh Ewald: An N·log(N) method for Ewald sums in large systems. *The Journal of Chemical Physics* **1993,** *98*, 10089-10092.
11. Nosé, S.; Klein, M. L., Constant pressure molecular dynamics for molecular systems. *Molecular Physics* **1983,** *50*, 1055-1076.
12. Parrinello, M.; Rahman, A., Polymorphic transitions in single crystals: A new molecular dynamics method. *Journal of Applied Physics* **1981,** *52*, 7182-7190.
13. Goga, N.; Rzepiela, A. J.; de Vries, A. H.; Marrink, S. J.; Berendsen, H. J. C., Efficient Algorithms for Langevin and DPD Dynamics. *Journal of Chemical Theory and Computation* **2012,** *8*, 3637-3649.
14. Beutler, T. C.; Mark, A. E.; van Schaik, R. C.; Gerber, P. R.; van Gunsteren, W. F., Avoiding singularities and numerical instabilities in free energy calculations based on molecular simulations. *Chemical Physics Letters* **1994,** *222*, 529-539.
15. NosÉ, S., A molecular dynamics method for simulations in the canonical ensemble. *Molecular Physics* **2002,** *100*, 191-198.
16. Hoover, W. G., Canonical dynamics: Equilibrium phase-space distributions. *Physical Review A* **1985,** *31*, 1695-1697.
17. Nikitin, A.; Wang, F., Simulation of Linear and Cyclic Alkanes with Second-Order Møller–Plesset Perturbation Theory through Adaptive Force Matching. *Journal of Chemical Theory and Computation* **2024,** *20*, 5241-5249.
18. Zhang, Y.; Otani, A.; Maginn, E. J., Reliable Viscosity Calculation from Equilibrium Molecular Dynamics Simulations: A Time Decomposition Method. *Journal of Chemical Theory and Computation* **2015,** *11*, 3537-3546.
19. Allen, M. P.; Tildesley, D. J., Computer Simulation of Liquids. Oxford University Press: 2017. https://doi.org/10.1093/oso/9780198803195.001.0001.
20. Rowlinson, J. S.; Widom, B., In *Molecular theory of capillarity*. Mineola, N.Y. : Dover Publications: New York (State), United States, 2002.
21. Hu, H.; Wang, F., The liquid-vapor equilibria of TIP4P/2005 and BLYPSP-4F water models determined through direct simulations of the liquid-vapor interface. *The Journal of Chemical Physics* **2015,** *142*, 214507.
22. Wegner, F. J., Corrections to Scaling Laws. *Physical Review B* **1972,** *5*, 4529-4536.
23. Hu, H.; Ma, Z.; Wang, F., Chapter Two - On the Transferability of Three Water Models Developed by Adaptive Force Matching. In *Annual Reports in Computational Chemistry*, Wheeler, R. A., Ed. Elsevier: 2014; Vol. 10, pp 25-43.
